# Supplementary material for: Multi-modal Monte Carlo MRI simulator of tissue microstructure
Source: Imaging Neurosci (Camb). 2026 Mar 25;4:IMAG.a.1177. doi: 10.1162/IMAG.a.1177 (PMC7618918; doi:10.1162/IMAG.a.1177)
Supplement: Supplementary Material [file IMAG.a.1177_supp.pdf]

Supplementary Information

S1. Determining the simulations timesteps

To accurately handle all of the features discussed in the main paper's theory section there are many constraints on the timestep of the simulator. These constraints include those imposed by the sequences, by the geometry, and by having a sufficient rate of collisions (i.e., short enough timestep) to accurately model permeability, surface relaxation, and magnetisation transfer.

If the timestep is too long for any of these constraints this could bias the final results. All of these constraints could be satisfied simply by using a very short timestep, which would be very computationally expensive. Here we discuss the algorithm used in the simulator to select the maximum timestep that can be used to obtain accurate simulation results with minimal computational costs.

Firstly, we split the total sequence (or multiple sequences) based on the start or end of any RF pulses or building blocks used in the sequence definition, as well as the timings of any instantaneous RF pulses, gradients, or readouts. At this stage we also ensure that within each period the gradient can be approximately described as changing linearly. This ensures that during any timestep the sequence is relatively homogeneous (e.g., there is a pulse active or there isn't) and that all of the instantaneous events are at the edges of any timestep.

Secondly, we iterate through each of the time periods from the split defined above and for each determine whether it should be split up any further. This is done by determining at each time period the maximum timestep $\tau_{\max}$. The total time period is then split up in uniform steps, where each step duration is shorter than $\tau_{\max}$.

The maximum timestep $\tau_{\max}$ is set to the minimum of various terms. The constants in front of each term have been empirically determined as illustrated in Figure S1 and Figure S2:

1. $0.03\ l^2/D$, where l is the minimum size scale of the obstructions included in the tissue microstructure phantom and D is the diffusivity. This reflects that for timesteps larger than this the ability of spins to travel around these obstructions is reduced (i.e., the tortuosity becomes timestep-dependent). The value of 0.03 is set based on simulations of this tortuosity (Figure S1). It also ensures that within compartments it is not consistently the same spin that undergoes all the collisions, but that instead the collision rate is equally shared across all spins (not shown). For cylinders and spheres the size scale is set to the radius, and for walls, to the distance between neighbouring walls. For meshes it is set by estimating the equivalent radius based on the local curvature of the mesh.
2. $0.5\ \theta_{\text{perm}}^{-2}$, which ensures that the permeability is accurately modelled (Figure S2B).
3. $0.01\ \theta_{\text{relax}}^{-2}$, which ensures that the surface relaxation is accurately modelled (Figure S2C).

4. $0.01 \left(\frac{\theta_{\text{transfer}}}{\theta_{\text{bound}}}\right)^2 \frac{D}{\pi}$, which ensures that the transition from free to bound states is accurately modelled (Figure S2D).
5. $0.1\,\theta_{\text{transfer}}$, which ensures that the same spins that have just been released from the bound state can return in a time short compared with the bound spin dwell time (θ_{transfer}) to get bound again at the same surface.
6. $\left(\frac{10^{-4}}{D\,\gamma^2 G^2}\right)^{\frac{1}{3}}$, where γ is the gyromagnetic ratio and G is the sequence gradient strength. This ensures that a spin taking a typical step size $l \approx \sqrt{D\tau}$ given the diffusivity and timestep will not move so much that the phase accumulation during that timestep would be very different at the beginning or end of the timestep (i.e., $\gamma G l \tau \ll 1$).

Note that the final constraint depends on the sequence, so the maximum timestep can be different at different times during the sequence evolution.

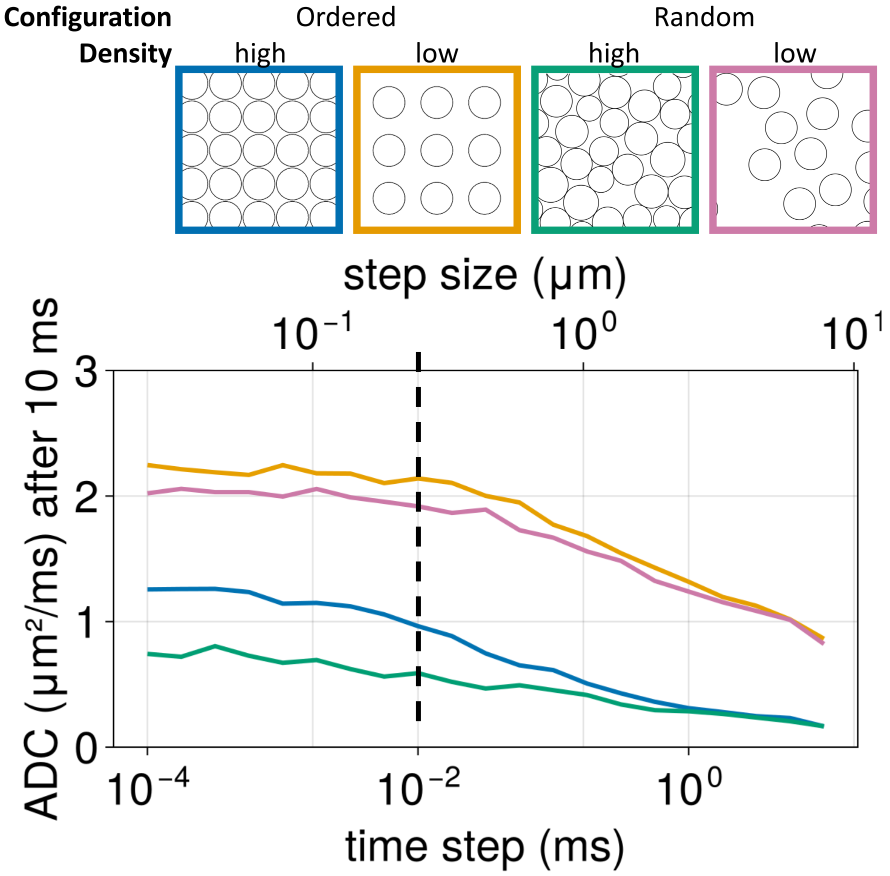

Figure S 1 Constraint on the timestep of the simulator in order to accurately model the tortuosity in the extra-cellular space. We test this case by considering various cylinder ($r = 1\mu$m) packing configurations with different levels of orderedness and density (top). The intrinsic diffusivity is 3 µm/ms², however this diffusivity is reduced orthogonal to the axons to some apparent diffusion coeffecient (ADC) due to the tortuosity of spins trajectories being hindered by the cylinders. For all simulated packing configurations we find that the diffusivity of spins orthogonal to these cylinders becomes timestep-independent for timesteps shorter than 0.01 ms. This can be extended to different radii and intrinsic diffusivities as $0.03\; l^2/D$, where l is the cylinder radius and D is the diffusivity.

Figures S2 compares three different methods to make the permeability, surface relaxation, and magnetisation transfer timestep-independent. For all metrics these three methods

converge for small timesteps, however we want to adopt the method that retains same result the longest as the timestep increases. For surface relaxation and magnetisation transfer this is the logarithmic adjustment (orange). For permeability this is also a logarithmic adjustment, but with the addition of a Bessel function (green). The result of these simulations is also used to set the maximum timestep allowed to accurately model these effects as listed above.

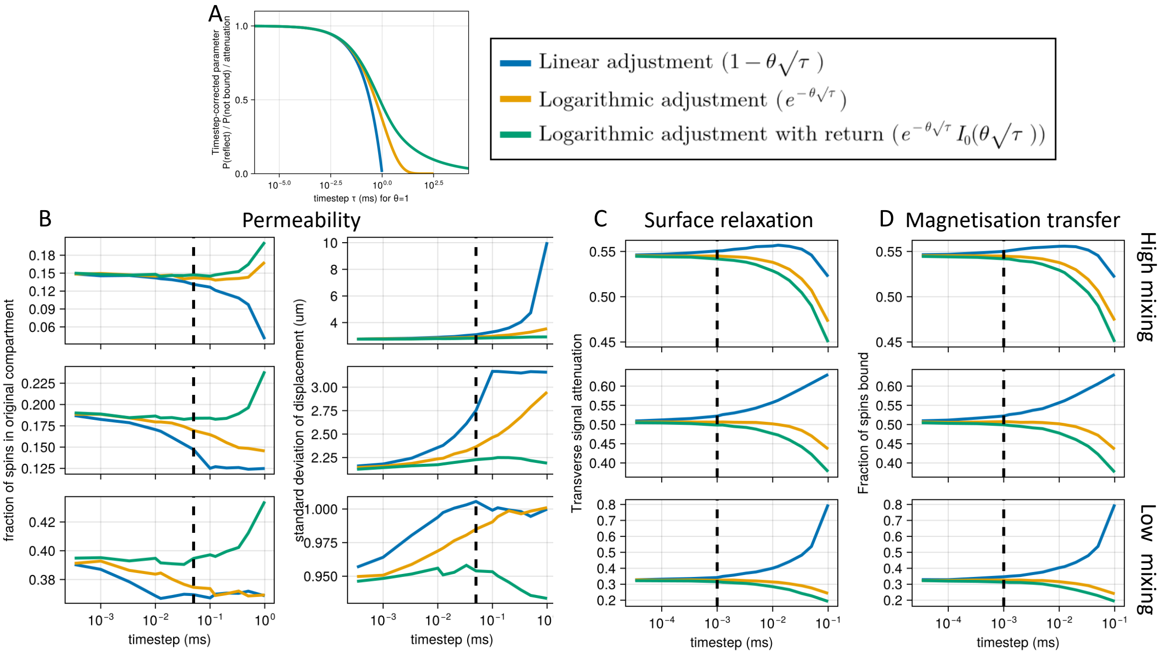

Figure S 2 Three different methods to correct for the timestep-dependence of the number of collisions (A) and how they affect the permeability (B), surface relaxation (C), and magnetisation transfer (D) in a simple toy example. This toy example considers 1-dimensional diffusion with obstructions regularly spaced every 1 µm. These obstructions may be permeable (B), have surface relaxation (C), or magnetisation transfer to a bound pool (D). For permeability we compare 2 metrics (fraction of spins in origin compartment and standard deviation of the displacement). For the other parameters we only consider a single metric (signal attenuation for surface relaxation and fraction of spins transferred from free to bound pool for magnetisation transfer). In B-D the top row shows an example for well-mixed compartments, while at the bottom the exchange/relaxation/transfer rates are short compared with the mixing times. The first correction method (blue) is derived from the naïve assumption that as the rate of collision doubles due to timestep changes, the effect of each collision should be halved. The second (orange) makes the same assumption, but halves the effect size in logarithmic space rather than linear space, which is appropriate for the surface relaxation and magnetisation transfer (C, D). Finally, the third (green) adds the Bessel function to take into account that two instances of the spin passing through a permeable membrane will effectively cancel each other out (B). The black dashed lines show the maximum timestep adopted by default in the simulator.

S2. Timestep-dependent collision rate

Here we derive the collision rate of spins with the surface in the simulations. This collision rate is crucial to correctly derive the exchange rate, surface relaxation, and magnetisation transfer rate within the simulator.

We will first derive this collision rate for the case of a single infinite wall before arguing why this result is more generally valid. In the case of the infinite wall, we only have to consider the position along a single dimension, namely along the wall normal. We refer to this dimension as x. The probability of a spin being between positions x and $x + dx$ is given by the line density λ multiplied with dx.

The probability of any random spin at distance x hitting the wall in the next timestep (with step size $l = \sqrt{2D\tau}$) is given by:

$$P(\text{hit}|x) = 1 - \Phi\left(\frac{x}{l}\right) = 1 - \Phi\left(\frac{x}{\sqrt{2D\tau}}\right) \tag{S1}$$

Where Φ is the cumulative distribution of the normal function:

$$\Phi(t) = \frac{1}{\sqrt{2\pi}} \int_{-\infty}^{t} e^{-\frac{t'^2}{2}} dt' \tag{S2}$$

Using this we can compute the total number of spins hitting the wall from one side as

$$\begin{aligned} N_{\text{hit}} &= \int_0^\infty P(\text{hit}|x)\, \lambda dx \\ &= \lambda \int_0^\infty 1 - \Phi\left(\frac{x}{\sqrt{2D\tau}}\right) dx \\ &= \lambda \sqrt{\frac{D\tau}{\pi}} \end{aligned} \tag{$S3$}$$

We can convert this to a finite patch of the wall with surface area S by using $\lambda = \rho S$, where ρ is the volumetric density:

$$N_{\text{hit}} = \rho S \sqrt{\frac{D\tau}{\pi}}, \tag{S4}$$

Finally, we consider that the spins are within a large voxel with volume V and total number of spins of N_{spins}:

$$\frac{N_{\text{hit}}}{N_{\text{spins}}} = \frac{S}{V} \sqrt{\frac{D\tau}{\pi}}, \tag{S5}$$

If this is the number of spins hitting the wall during each timestep τ, the collision rate r_c is given by:

$$r_c = \frac{1}{\tau} \frac{N_{\text{hit}}}{N_{\text{spins}}} = \frac{S}{V} \sqrt{\frac{D}{\pi\tau}} \tag{S6}$$

The above derivation assumes that spins can hit the wall from infinitely far (as we integrate to infinity). If there are other obstructions in the way then this will not hold. However, the number of spins prevented from hitting the wall by other obstructions, will be exactly replaced by the number of spins hitting the wall after bouncing of these other obstructions. So, the total rate of collisions is unaffected by the presence of other obstructions. A similar logic can be used to conclude that both permeability and magnetisation transfer also do not affect the collision rate.
